# Blame framing and prior knowledge influence moral judgments for people involved in the Tulsa Race Massacre among a combined Oklahoma and UK sample

**DOI:** 10.3389/fpsyg.2024.1251238

**Published:** 2024-02-21

**Authors:** Justin D. Durham, Adon F. G. Rosen, Scott D. Gronlund

**Affiliations:** Department of Psychology, University of Oklahoma, Norman, OK, United States

**Keywords:** Tulsa Race Massacre, blame framing, prior knowledge, moral cognition, mixed-effects modeling

## Abstract

**Introduction:**

How an event is framed impacts how people judge the morality of those involved, but prior knowledge can influence information processing about an event, which also can impact moral judgments. The current study explored how blame framing and self-reported prior knowledge of a historical act of racial violence, labeled as Riot, Massacre, or Event, impacted individual’s cumulative moral judgments regarding the groups involved in the Tulsa Race Massacre (Black Tulsans, the Tulsa Police, and White Tulsans).

**Methods and results:**

This study was collected in two cohorts including undergraduates attending the University of Oklahoma and individuals living in the United Kingdom. Participants were randomly assigned to a blame framing condition, read a factual summary of what happened in Tulsa in 1921, and then responded to various moral judgment items about each group. Individuals without prior knowledge had higher average Likert ratings (more blame) toward Black Tulsans and lower average Likert ratings (less blame) toward White Tulsans and the Tulsa Police compared to participants with prior knowledge. This finding was largest when what participants read was framed as a Massacre rather than a Riot or Event. We also found participants with prior knowledge significantly differed in how they made moral judgments across target groups; those with prior knowledge had lower average Likert ratings (less blame) for Black Tulsans and higher average Likert ratings (more blame) for White Tulsans on items pertaining to causal responsibility, intentionality, and punishment compared to participants without prior knowledge.

**Discussion:**

Findings suggest that the effect of blame framing on moral judgments is dependent on prior knowledge. Implications for how people interpret both historical and new events involving harmful consequences are discussed.

## 1 Introduction

The Tulsa Race Massacre is one of the worst acts of racial violence in American history (Halliburton, 1972). The Tulsa Race Massacre was referred to as the Tulsa Race Riot until 2018, when the name was changed by the *Oklahoma State Tulsa Race Massacre Commission* because “Massacre” more accurately represented what happened and, according to Oklahoma State Senator Kevin Matthews, was an effort to, “heal wounds of people here now” (2 News Oklahoma, 2018; Krehbiel, 2018). The eponymous reframing underscores the necessity to accurately label caused-harm in a way that is consistent with the nature of the event. To emphasize veracity and justice, it is important that the framing of historical events reflect the experiences of the people involved. The purpose of this study was to explore how the blame framing of what happened in Tulsa, and people’s prior knowledge of the Tulsa Race Massacre, influenced judgment ratings about the moral behavior of the groups involved.

Our study manipulated whether a summary referred to what happened in Tulsa in 1921 as a Riot, a Massacre, or an Event. The manipulation of the name change afforded insights into how individuals make judgments of causal responsibility, blame punishment, and related moral judgments. The framing and semantics of the label, “Riot”, signaled that Black Tulsans were responsible for what happened and should result in participants attributing negative moral judgments toward Black Tulsans and positive moral judgments toward White Tulsans. Conversely, framing what happened as a “Massacre” should result in participants attributing positive judgments toward Black Tulsans and negative judgments toward White Tulsans. The use of the label “Event”, which is ambiguous in terms of meaning, served as a control condition of neutral responsibility. Moreover, whenever we mention predictions about White Tulsans, we expect something similar to happen regarding moral judgments toward the police.

Many people from the United States likely have some prior knowledge about, and thus, a better understanding of, the Tulsa Race Massacre, compared to people from other parts of the world. Having prior knowledge likely gives individuals a better understanding of what happened in Tulsa and who was responsible. Prior knowledge should result in more negative judgments toward White Tulsans and lower negative judgments of Black Tulsans. In contrast, not having prior knowledge might result in people having lower negative judgments of White Tulsans and more negative judgments toward Black Tulsans.

We collected and combined data for this study across two cohorts, one consisting of undergraduates from the University of Oklahoma (OU) and one sampled from the general public in the United Kingdom (UK). We collected self-reported prior knowledge regarding the events in Tulsa as a dichotomous variable (i.e., yes or no) and reasoned that most individuals from OU possessed some prior knowledge while a majority of the UK sample would not. In sum, we explored how the framing of the label, and an individual’s knowledge of the events in Tulsa, influenced responses on moral judgment items (i.e., causal responsibility, blame, punishment, etc.) for the groups involved (i.e., Black Tulsans, Tulsa Police, and White Tulsans).

### 1.1 Blame framing

People’s judgments or explicit attitudes about the moral evaluations individuals make about people (including oneself), places, things, and ideas are driven by their emotions and unique experiences rather than solely by cognitive reasoning. Cognitive neuroscience research supports the claim that reasoning is important, but preconscious emotional processes also influence moral judgments (Greene and Haidt, 2002). A moral judgment of an agent’s intention to commit harm is related to how they attribute blame for the harm. Some research has shown that moral judgments of harmful consequences are more sensitive to changes in valence than intentionality (Guglielmo and Malle, 2010). The context of a scenario also can impact moral judgments for harmful consequences (Cushman, 2008; Schein, 2020) and the cognitive process by which individuals assign blame (Malle et al., 2014; Guglielmo, 2015). We were primarily interested in exploring whether the connotation of the label (i.e., Riot, Massacre, and Event) influenced how blame and related moral judgments were assigned to each group.

Research has found that subtle changes in how news events are described can influence perceptions and how people make causal attributions. For example, Knobloch-Westerwick et al. (2008) found that when an individual’s or organization’s actions are described using active voice, that individual or organization is perceived as having caused the event relative to when passive voice is used. In a second study, they found that the more causal responsibility participants assigned to an agent, the less support there was for that agent’s view. Fausey and Boroditsky (2010) examined how agentive and non-agentive language can shape how people attribute blame and financial liability to individuals involved in accidents. They found that participants attributed greater blame and harsher punishment when using agentive language compared to non-agentive language, even when people had established knowledge and visual information about the event. Although our study does not manipulate verbalizations, but rather only the label, of the Tulsa Race Massacre, this research demonstrates how subtle changes in language can influence blame and punishment.

In contrast, different results were possible in the present study because it involved a historical account of racial violence that resulted in negative consequences. In general, participants should be motivated to control their biases and should have more positive moral judgments for Black Tulsans compared to White Tulsans. We expected this motivation would be enhanced for participants with prior knowledge. This research also suggests that those that read about what happened framed as a Massacre should make more negative judgments toward White Tulsans and less negative judgments toward Black Tulsans, compared to if what happened was framed as a Riot. Furthermore, we expected less negative judgments for Black Tulsans when what happened was framed as a Massacre and more negative judgments for Black Tulsans when what happened was framed as a Riot.

### 1.2 Prior knowledge

Moral judgments also may vary depending on prior knowledge about what happened. Individuals actively search for meaning and construct inferences from a story based on their prior knowledge and unique experiences (Pressley and Afflerbach, 2012). Social and moral knowledge are guided by an individual’s schema – cognitive templates or general knowledge structures – and their attitudes (e.g., likeability) toward people, places, and events, which also influence information processing (Bartlett, 1932; Narvaez, 2002; Carlston, 2010). People extract the important ideas from a story, which vary depending on one’s perspective (e.g., whether one is told prior to reading a story that they are a robber or a homebuyer who is walking through a house (Anderson and Pichert, 1978). This suggests that whether an event is framed as a Massacre (which implies violence against Black Tulsans) or a Riot (which implies that both groups share blame) should have implications for what one takes from reading about the events in Tulsa, especially for those with limited to no prior knowledge.

Zaromb et al. (2014) had young and older adults recall the 10 most important events that occurred during the Civil War, World War II, and the Iraq War, as well as evaluate the emotional valence, relative importance, and their level of knowledge for each event. They found that collective memories differ depending on whether the events are experienced personally or learned from historical sources. By extension, this suggests that those with prior knowledge for the Tulsa Race Massacre should possess a stronger mental representation for what happened and should make judgments that are consistent with a collective memory for the events in Tulsa. Overall, this should result in more blame toward White Tulsans and less blame toward Black Tulsans for individuals with prior knowledge compared to those without prior knowledge.

### 1.3 Purpose

The present study examined whether the labeling of a historical event, together with prior knowledge about the event, influenced moral judgments (e.g., causal responsibility, blame, punishment) about the parties involved. In our analysis, we first examined the highest-ordered interactions that included either blame framing and prior knowledge. These higher order interactions were then explored in a progressive stepwise fashion. Our research questions and hypotheses included:

A Do the average Likert ratings for target groups by blame framing differ across prior knowledge (Framing x Knowledge × Target)?

*Hypothesis 1:* We expected to see differences in prior knowledge across blame framing whereby participants without prior knowledge make higher average Likert ratings for Black Tulsans and lower average Likert ratings for White Tulsans across blame framing compared to individuals with prior knowledge, but we expected this difference to be largest when what happened was framed as a Massacre.

B Do average Likert ratings for target groups differ across blame framing (Framing × Target)?

*Hypothesis 2*: Massacre is expected to result in lower average Likert ratings for Black Tulsans and higher average Likert ratings for White Tulsans compared to Riot.

C Do average Likert ratings judgments for target groups differ across individuals’ prior knowledge of the Tulsa Race Massacre (Knowledge × Target)?

*Hypothesis 3*: Individuals with prior knowledge should have higher average Likert ratings for White Tulsans and lower average Likert ratings for Black Tulsans.

## 2 Methods

### 2.1 Participants

Data for this experiment were collected in two cohorts, an OU sample and a UK sample. Participants in the OU sample received course credit in exchange for completing the study whereas participants in the United Kingdom sample received compensation for completing the study. The OU cohort was comprised of 190 undergraduate participants and were collected from February 2021 to March 2021 using Qualtrics. From the 190 responses, six were dropped due to incomplete cases or selecting not to allow the experimenters to use their data, resulting in 184 (108 Females, 74 Males, and 2 Prefer not to answer; majority were aged 18–24) total responses. Responses from 347 participants living in the United Kingdom were collected in August 2021 using SurveyMonkey. Out of 347 completed cases, 225 were removed due to incomplete cases, selecting not to allow the experimenters to use their data, and/or completing the study quicker than 5 min or slower than 90 min. This resulted in a sample of 122 (67 Female, 54 Male, 1 Prefer not to answer; over half were less than 45 years of age) for final analyses. Ethnicity was only recorded in the United Kingdom sample (Asian/Asian British = 14 (11%), Black/African/Caribbean/Black British = 6 (5%), Mixed/Multiple ethnicities = 6 (5%), White = 94 (77%), Prefer not to answer = 1 (<1%), Other = 1 (<1%)). The combined dataset resulted in a total of 306 participants. The study protocol was approved by the University of Oklahoma’s Institutional Review Board and all participants received informed consent before beginning the study. All methods were performed under the relevant guidelines and regulations.

### 2.2 Experimental design

This study included a one-way between-subjects experimental design with three levels of framing condition. Participants were randomly assigned to only one framing condition. Data collection occurred across two cohorts.

### 2.3 Materials

This study consisted of a summary article and a series of judgment items. A factual summary of the Tulsa Race Massacre was used as the article stimulus. The authors developed and adapted the stimulus from Ellsworth (2001, 2010) into an 827-word factual summary. The summary differed in the blame framing the participants received: Riot, Massacre, or Event. The title of the article summary included the manipulated blame framing and the label was presented a total of nine times within the summary (see Supplementary information). Nine judgment items related to various dimensions of moral cognition including causal responsibility, blame, and punishment (e.g., rate the degree of blame attributable to each group, see Supplementary information for full list of judgment items), were developed for the study. These judgment items were adapted from previous articles testing for how culpability and intentionality are associated with judgments of blame and punishment (see Alicke, 2000; Knobe, 2003; Cushman, 2008). Participants in Oklahoma completed the study on Qualtrics and participants in the United Kingdom completed the study on SurveyMonkey.

### 2.4 Procedure

Participants in both study cohorts completed the study online and were instructed to read a summary article and respond to judgment items about what they read. After reading the summary, participants answered nine judgment items, all items presented in the same order. Participants also completed demographic items before being debriefed on the nature of the study and the history of the Tulsa Race Massacre. Finally, participants completed items on whether they had prior knowledge of the Tulsa Race Massacre (Yes or No) and whether to allow the researchers to use their data (Yes or No).

Both cohorts completed the study in a conceptually identical manner but differed in three ways. First, the OU sample instructed participants they would have 6.5 min to read the article and included a countdown timer that automatically advanced the study once the timer ended. This was done to try to control participants’ learning of the material. The UK sample did not have these time constraints (due to software limitations) and participants could advance when ready. Second, the UK sample included a condensed set of judgment items compared to the OU sample. The current analysis included only those judgment items that were in both samples. Third, participants in the UK sample received payment through SurveyMonkey for completing the study while participants in the OU study received course credit.

## 3 Results

Data were prepared for a mixed-effects regression analysis. Fixed-effect predictors included: the blame framing participants were assigned to, the judgment items, target group, whether the participant had prior knowledge of the event, and the participant’s cohort. The participant was included as a random effect. The predictors of interest included the assigned blame framing, which was coded as a 3-level factor (Riot, Massacre, Event), the judgment items (e.g., rate the degree of blame attributable to each group’s behavior), and the target group (Black Tulsans, Police Officers, and White Tulsans), as well as prior knowledge, which was coded as a dichotomous factor (Yes/No). All models controlled for the participant’s cohort.

### 3.1 Descriptive statistics

Most participants in the OU cohort (*n* = 184) had prior knowledge about the Tulsa Race Massacre before taking the study (*n* = 137, 74%) whereas few participants in the UK cohort (*n* = 122) had prior knowledge (*n* = 33, 27%). When collapsed across cohorts, there was a relatively similar number of participants with prior knowledge and no prior knowledge across blame framing. No significant difference in the proportions of sample size at each factor level of cohort, blame framing, and prior knowledge were observed.

### 3.2 Mixed-effects models

Mixed effect models were employed in order to control for Type-1 error; further correction for Type-1 errors was made when parceling these interactions. Mixed-effects models explored differences in the Likert rating (1–7) participants attributed across nine judgment items, target group, blame framing, and participant’s prior knowledge. The primary dependent variable for the mixed-effects results described is the average Likert rating participants assigned across all items. Higher Likert ratings indicated more negative judgments (i.e., degree of causal responsibility, blame, punishment) attributed to a target group across other factors while lower Likert ratings indicated less negative judgments.

We explored the relationship between these variables in our full model by first examining the highest-order interaction effects containing blame framing and/or prior knowledge and then subsequently explored these relationships in reduced models. Up to all 4-way interactions were included in the model (see Supplementary information for full model ANOVA table):

$\begin{array}{l} \mathrm{Average}\;\mathrm{Judgment}\sim \left(\begin{array}{l} \mathrm{Framing}+\mathrm{Knowledge}\\ +\mathrm{Target}+\mathrm{Item} \end{array}\right)\\ \;4+\mathrm{Cohort}+\left(1|\mathrm{Subject}\right)\text{.} \end{array}$

Interactions were explored in a hierarchical fashion: the highest order interaction was explored, and then separate models were trained within conditions of the highest order interaction. Multiple comparison tests employed false discovery rate (FDR) correction (Benjamini and Hochberg, 1995).

#### 3.2.1 Blame framing × prior knowledge × target group

**Figure 1:**
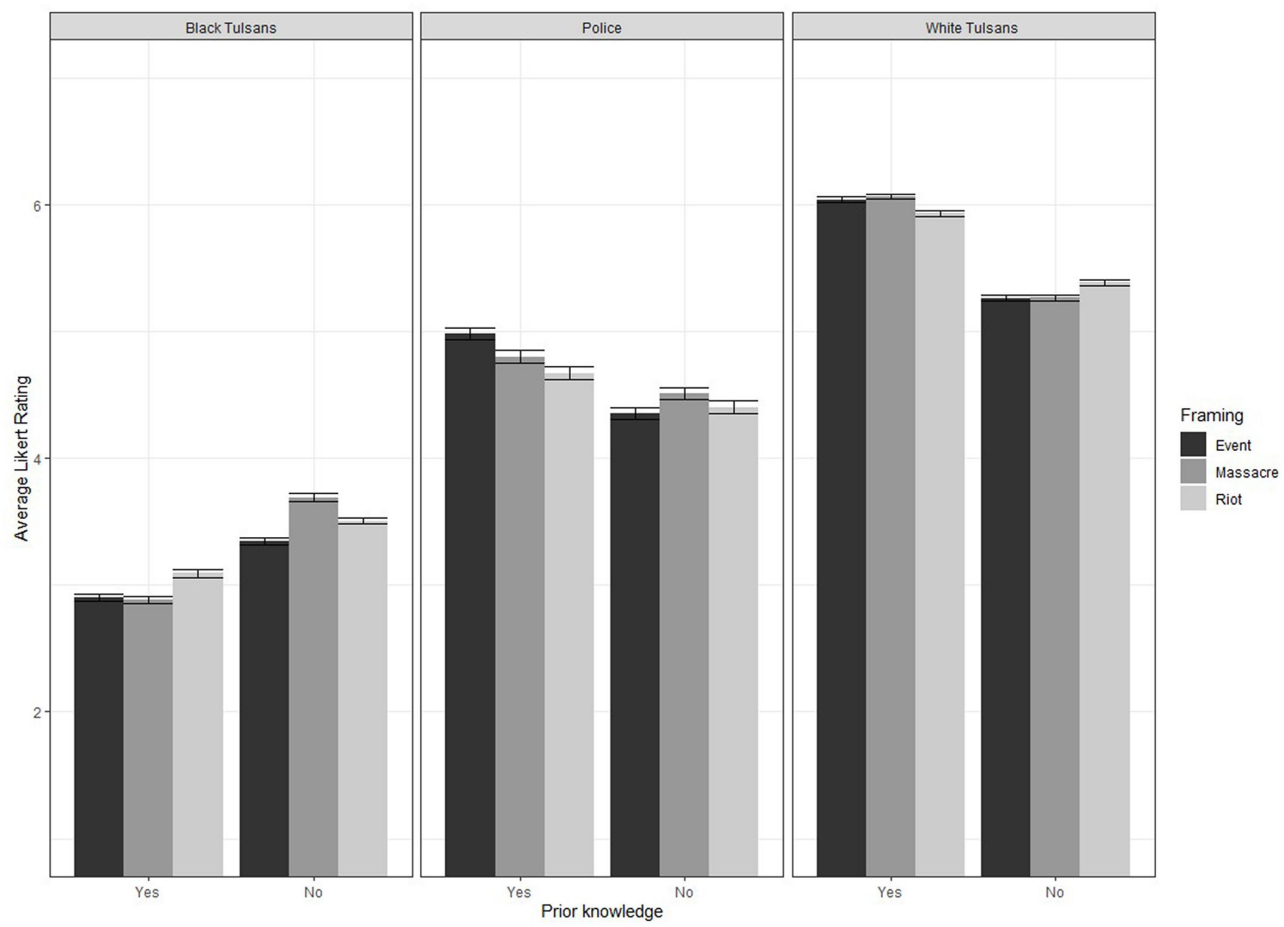
Three-way interaction of framing × prior knowledge × target group on average Likert rating. Black bars indicate average Likert ratings for Event blame framing, dark gray bars indicate average Likert ratings for Massacre blame framing, and light gray bars indicate average Likert ratings for Riot blame framing. Error bars indicate standard error of the mean.

Results for our full model found a three-way interaction for Blame Framing × Prior Knowledge × Target Group (*F*(4, 7,800) = 3.46, *p* = 0.01) indicating the average Likert rating for target groups differed by blame framing and prior knowledge. This effect appeared to be driven by those without prior knowledge, who had higher average Likert ratings (i.e., more blame) for Black Tulsans (*t*(299) = 2.23, *p* < 0.05) and lower average Likert ratings for White Tulsans (*t*(299) = −3.06, *p* < 0.005) and the Police (*t*(299) = −3.22, *p* < 0.005; see Figure 1). However, these trends were moderated by the blame framing a participant was assigned. For example, individuals with prior knowledge in the Riot framing yielded higher average Likert ratings for Black Tulsans compared to the Event and Massacre framing (*t*(167) = 2.04, *p* < 0.05), while for those without knowledge the Massacre framing yielded a significantly higher average Likert rating than the Event and Riot framing (*t*(133) = 2.06, *p* < 0.05). Similar discrepancies were observed when participants were assigning ratings to the Police; when participants possessed prior knowledge the Event framing received significantly higher ratings than the Massacre and Riot conditions (*t*(167) = 2.70, *p* < 0.01), while no significant differences were observed across conditions when participants did not have prior knowledge. Finally, no significant differences were observed across conditions, within prior knowledge groups, for the White Tulsans. This finding provides support for our hypothesis that participants without prior knowledge will make more negative judgments for Black Tulsans and less negative judgments for White Tulsans across framing compared to individuals with prior knowledge. This finding also was largest in the Massacre framing.

#### 3.2.2 Prior knowledge × target group × judgment item

**Figure 2:**
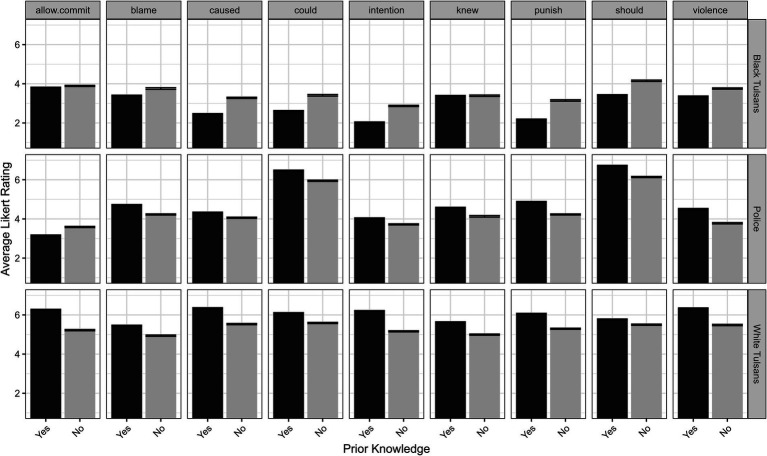
Three-way interaction of prior knowledge × target group × judgment item on average Likert rating. Black bars indicate average Likert rating for participants with prior knowledge and gray bars indicate average Likert rating for participants without prior knowledge. Error bars indicate standard error of the mean.

A significant effect between Prior Knowledge × Target Group × Judgment Item (*F*(16, 7,800) = 3.98, *p* < 0.005) was also observed indicating that average ratings for judgment items differed across target group and prior knowledge. Accordingly, target differences were explored within each judgment item across prior knowledge groups. For Black Tulsans, five of the nine items displayed significant prior knowledge differences in which participants with prior knowledge had lower average Likert ratings of causal responsibility (β_PK=NO_ = 0.93, *t*(301) = 4.71, *q* < 0.005), intentionality (β_PK=NO_ = 0.93, *t*(301) = 4.90, *q* < 0.005), punishment (β_PK=NO_ = 1.00, *t*(301) = 4.74, *q* < 0.005), should have prevented (β_PK=NO_ = 0.90, *t*(301) = 3.50, *q* < 0.005), and could have prevented (β_PK=NO_ = 0.92, *t*(301) = 3.96, *q* < 0.005), compared to participants without prior knowledge. For White Tulsans, seven out of the nine items displayed significant prior knowledge differences in which participants with prior knowledge had higher average Likert ratings for violence committed (β_PK=NO_ = −0.60, *t*(301) = −3.63, *q* < 0.005), causal responsibility (β_PK=NO_ = −0.66, *t*(301) = −4.07, *q* < 0.005), intentionality (β_PK=NO_ = −0.95, *t*(301) = −4.82, *q* < 0.005), punishment (β_PK=NO_ = −0.71, *t*(301) = −3.40, *q* < 0.05), allowed rather than committed what happened (β_PK=NO_ = −0.57, *t*(301) = −2.76, *q* < 0.05), knew what would happen (β_PK=NO_ = −0.64, *t*(301) = −2.91, *q* < 0.05), and could have prevented what happened (β_PK=NO_ = −0.40, *t*(301) = −2.24, *q* < 0.05), compared to participants without prior knowledge. For Police, three of the nine items displayed significant prior knowledge differences in which participants with prior knowledge had higher average Likert ratings for violence committed (β_PK=NO_ = −0.62, *t*(301) = −3.00, *q* < 0.05), punishment (β_PK=NO_ = −0.70, *t*(301) = −3.01, *q* < 0.05), and should have prevented what happened (β_PK=NO_ = −0.30, *t*(301) = −2.10, *q* < 0.05), compared to participants without prior knowledge (see Figure 2).

**Figure 3:**
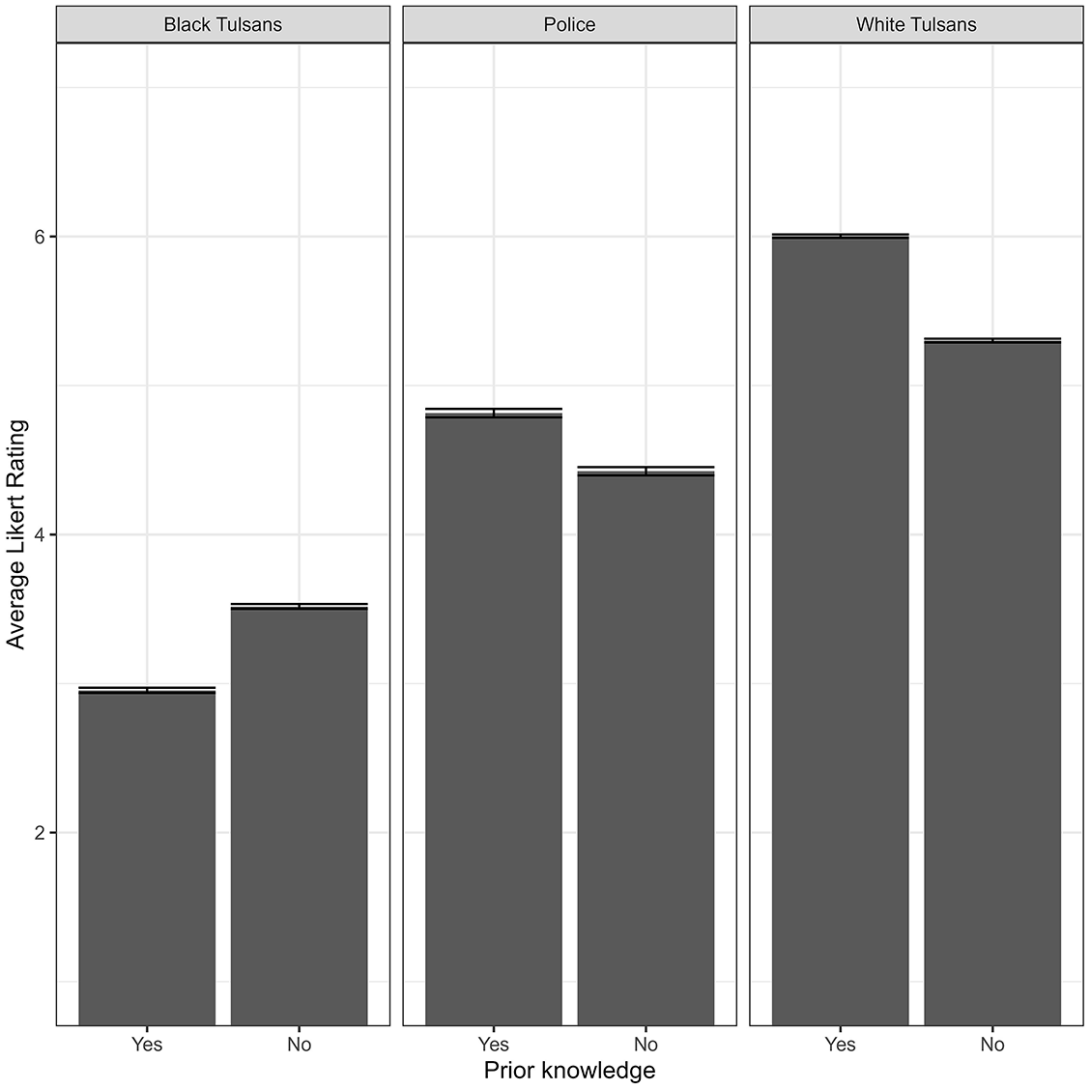
Two-way interaction of prior knowledge × target group on average Likert rating. Error bars indicate standard error of the mean.

We further examined this relationship by exploring two-way interactions between prior knowledge and the target group. We found a significant two-way interaction, (*F*(2, 7,800) = 117, *p* < 0.05). Pairwise comparisons further examined differences between prior knowledge within each target group. Individuals with prior knowledge made significantly higher average Likert ratings for White Tulsans (*β*_PK=NO_ = −0.61, *t*(301) = −4.90, *q* < 0.001), and Tulsa Police (*β*_PK=NO_ = −0.35, *t*(301) = −3.13, *q* < 0.005); average Likert ratings were significantly lower for Black Tulsans (*β*_PK=NO_ = 0.64, *t*(301) = 4.58, *q* < 0.001) compared to individuals without prior knowledge (see Figure 3).

## 4 Discussion

The purpose of this study was to examine the effect that blame framing and prior knowledge has on an individual’s moral judgments for the groups involved in the Tulsa Race Massacre. Participants were randomly assigned to a blame framing condition (Event, Massacre, or Riot), read a summary of the events that took place in Tulsa in 1921, and then made moral judgments (including causal responsibility, blame, and punishment) about the groups involved (Black Tulsans, Tulsa Police, White Tulsans).We used an Oklahoma and international sample to examine how participants made moral judgments about the groups involved in this historical act of racial violence. We analyzed our data using mixed effects models, including fixed and random effects, to control individual variation in attributional patterns and to explore moral judgments across the cohort samples.

We found support for our main hypothesis showing a significant interaction between blame framing, prior knowledge, and target groups. Participants with prior knowledge attributed less negative judgments toward Black Tulsans and more negative judgments toward White Tulsans compared to participants without prior knowledge; these differences were larger in the Massacre framing relative to the Riot framing but were not statistically supported when using FDR corrections. Moreover, participants with prior knowledge showed greater variation in their average judgments to the target groups across blame framing compared to participants without knowledge. This interaction suggests that how people make moral judgments about the target groups across blame framing depends on their prior knowledge. Whereas the hypothesis that framing and target group interact on average Likert rating was not statistically supported, this interaction was potentially masked by prior knowledge.

Our results also showed a significant interaction for prior knowledge, target groups, and the judgment items on average Likert ratings. Participants with prior knowledge made less negative judgments for Black Tulsans on items involving the degree of causal responsibility, intentionality, punishment, should have prevented what happened, and could have prevented what happened, compared to participants without prior knowledge. Participants with prior knowledge had higher average Likert ratings for White Tulsans on items involving the degree of violence, intentionality, and allowing versus committing what happened, compared to participants without prior knowledge. This suggests that having prior knowledge affords individuals a different representation of what transpired and allows them to make moral judgments in a manner that is more consistent with what really happened. We also found participants with prior knowledge made significantly higher average Likert ratings for White Tulsans and Tulsa Police, and lower average Likert ratings for Black Tulsans, compared to individuals without prior knowledge. This supported our hypothesis that judgments for target groups differ by prior knowledge. These findings suggest that how participants morally judged the behavior of the target groups involved in the Tulsa Race Massacre depended on whether participants had prior knowledge. The fact that moral judgments for target groups was driven by participants’ prior knowledge has implications for how an individual’s understanding of an event influences their judgments about who caused harmful consequences, who is blameworthy, and who deserves punishment. Having adequate prior knowledge is relevant for learning and understanding harmful consequences, which, in the case of our study, impacted moral judgments.

Our analyses found that the effect of blame framing on moral judgments for target groups depended on participant’s prior knowledge for the Tulsa Race Massacre. Our study also has the added benefit of examining how people make moral judgments about a historical act of racial violence while also informing participants about the Tulsa Race Massacre. Nonetheless, this study has limitations that must be considered. First, we measured prior knowledge of the Tulsa Race Massacre as a dichotomous variable rather than in a continuous manner, and we did not collect details on the sources and accuracy of information acquired. Nevertheless, this single self-reported item clearly captures something relevant about an individual’s subjective knowledge for the Tulsa Race Massacre. However, if we had collected greater information on when, what, and how participants acquired knowledge about the Tulsa Race Massacre, we could have further explored whether there were differences in moral judgments for participants who had recently learned about the Tulsa Race Massacre in the media (e.g., HBO’s Watchmen) compared to those that acquired the knowledge in their primary or secondary education.

We collected data from a UK sample with the assumption that a large proportion of participants would not have prior knowledge about the Tulsa Race Massacre. However, there are likely differences between the two cohorts we did not account for in terms of factors such as age, education, and prior experiences with people of color, as well as, cultural factors such as perceptions regarding Black Lives Matter (BLM) and Diversity, Equity, and Inclusion (DEI). Although we did not seek to directly explore how individual and cultural factors impact how participants make moral judgments about the Tulsa Race Massacre, we may have confounded differences across prior knowledge. Research avenues that more directly examine how these factors influence moral judgments about social groups in various contexts would provide substantial benefits in better understanding how cultural and psychological processes influence moral judgments.

Second, our study did not find an effect of framing alone, suggesting that our manipulation may have been too subtle to produce a measurable effect on participants’ moral judgments. In line with previous research, a framing effect was observed, but it was limited to participants with prior knowledge. This is consistent with a recent meta-analysis showing that valence framing does impact moral judgments, albeit the magnitude of this effect is small (Cohen’s *d* = 0.22, McDonald et al., 2021). Our stimuli included a historical summary of the Tulsa Race Massacre without including any information about a groups’ desire to behave in a particular manner. If we had included additional language that aligned the caused harm with White Tulsan’s desires to commit the act, then we might have seen larger effects of framing. Finally, although our study randomly assigned participants to framing conditions, it may have benefited by incorporating additional methodology to control for additional sources of variability (i.e., pre-post design, repeated measures, or modeling techniques). Despite these limitations, our study found evidence that blame framing and prior knowledge together influence participant’s moral judgments for the Tulsa Race Massacre.

Future research should examine how people understand and make moral judgments about the groups involved in historical events. Conducting experiments to examine how labels influence moral judgments may be informative to institutions that need to decide how to label specific events, such as truth commissions (see Mosby and Millions, 2021). This study should encourage future researchers to examine knowledge with greater granularity. Conducting additional research to address these issues would not only be informative regarding how people understand and make judgments about well-known, historical events involving harmful consequences, but also about events yet to come.

## 5 Conclusion

This study explored the effects of blame framing on moral judgments for groups involved in the Tulsa Race Massacre; it also explored the effects of prior knowledge about the Tulsa Race Massacre among undergraduates at OU and people living in the UK. Results suggest that (a) responses to moral judgment items concerning each target group depended on participants’ prior knowledge, and (b) the effect of framing for judgments toward target groups differed by prior knowledge.

## Data availability statement

The datasets presented in this study can be found in online repositories. The names of the repository/repositories and accession number(s) can be found at: https://github.com/adrose/durhamTulsaProj.

## Ethics statement

The studies involving humans were approved by University of Oklahoma, Institutional Review Board. The studies were conducted in accordance with the local legislation and institutional requirements. The participants provided their written informed consent to participate in this study. Written informed consent was obtained from the individual(s) for the publication of any potentially identifiable images or data included in this article.

## Author contributions

JD: conceptualization, methodology, writing – original draft preparation, and analysis. AR: analysis, writing – review and editing. SG: conceptualization, supervision, writing – reviewing and editing. All authors contributed to the article and approved the submitted version.
