# Supplementary material for: Blame framing and prior knowledge influence moral judgments for people involved in the Tulsa Race Massacre among a combined Oklahoma and UK sample
Source: Front Psychol. 2024 Feb 21;15:1251238. doi: 10.3389/fpsyg.2024.1251238 (PMC10915277; doi:10.3389/fpsyg.2024.1251238)
Supplement: Supplementary file 1 [file Data_Sheet_1.PDF]

Supplementary Information

Data availability

The data and R code used for analysis are available on Github at

https://github.com/adrose/durhamTulsaProj.

Supplementary Materials S1

LABEL = Massacre, Riot, or Event

TULSA RACE *LABEL*

The Tulsa *Label* has continued to haunt Oklahomans for nearly 100 years. During the course of eighteen hours on May 31 and June 1, 1921, more than one thousand homes and businesses were destroyed, while credible estimates of deaths range from fifty to three hundred. By the time the *Label* ended, the city had been placed under martial law, thousands of Tulsans were being held under armed guard, and the state's second-largest African American community had been burned to the ground. One of a number of similar episodes nationwide, the *Label* occurred during an era of acute racial tensions, particularly in the matter of lynching. Such trends were mirrored both statewide and in Tulsa.

By early 1921, Tulsa was a modern city with a population of more than one hundred thousand. Most of the city's ten thousand African American residents lived in the Greenwood District, a vibrant neighborhood that was home to two newspapers, several churches, a library branch, and scores of Black-owned businesses. However, Tulsa was also a deeply troubled town. Crime rates were high, and the city had been plagued by vigilantism, including the August 1920 lynching, by a White mob, of a White teenager accused of murder. Newspaper reports confirmed that the Tulsa police had done little to protect the lynching victim, who had been taken from his jail cell at the county courthouse.

Eight months later an incident involving Dick Rowland, an African American shoe shiner, and Sarah Page, a White elevator operator, would set the stage for tragedy. While it is still uncertain precisely what happened in the Drexel Building on May 30, 1921, the most common explanation is that Rowland stepped on Page's foot as he entered the elevator, causing her to scream. The next day, however, the *Tulsa Tribune*, the city's afternoon daily newspaper, reported that Rowland, who had been picked up by police, had attempted to rape Page. By early evening there was talk of lynching on the streets of Tulsa.

Talk soon turned to action. By 7:30 p.m., hundreds of Whites had gathered outside the Tulsa County Courthouse, demanding that the authorities hand over Rowland, but the sheriff refused. At about 9 p.m., after reports of the dire conditions downtown reached Greenwood, a group of approximately twenty-five armed African American men, many of them World War I veterans, went down to the courthouse and offered their services to the authorities to help protect Rowland. The sheriff, however, turned them down, and the men returned to Greenwood. Stunned, and then enraged, members of the White mob then tried to break into the National Guard armory but were turned away by a handful of local guardsmen. At about 10 p.m., a false rumor hit Greenwood that Whites were storming the courthouse. This time, a second contingent of African American men, perhaps seventy-five in number, went back to the courthouse and offered their services to the authorities. Once again, they were turned down. As they were leaving, a White man tried to disarm a Black veteran, and a shot was fired. The ***Label*** began.

Over the next six hours, Tulsa was plunged into chaos. Furious fighting erupted along the Frisco railroad tracks, where Black defenders were able to hold off members of the White mob. By midnight, fires had been set along the edge of the African American commercial district. During the early hours of the conflict, local authorities did little to stem the growing crisis.

Indeed, shortly after the outbreak of gunfire at the courthouse, Tulsa police officers armed and deputized former members of the mob and instructed them to apprehend African Americans. Local units of the National Guard were mobilized, but they spent most of the night protecting a White neighborhood from a feared, but nonexistent, Black counterattack.

Shortly before dawn on June 1, thousands of armed Whites had gathered along the fringes of Greenwood. When daybreak came, they poured into the African American district. At least one machine gun was utilized, and some participants have claimed that airplanes were also used in the *Label*. Black Tulsans fought hard to protect their homes and businesses, with particularly sharp fighting occurring off of Standpipe Hill. In the end, they were simply outgunned and outnumbered. By the time that additional National Guard troops arrived in Tulsa at approximately 9:15 a.m. on the morning of June 1, most of Greenwood had already been put to the torch and the *Label* had ended.

Although Dick Rowland eventually was exonerated of any wrongdoing, an all-White grand jury blamed Black Tulsans for the lawlessness. However, no one was ever sent to prison for the murders and arson that occurred. Despite efforts by the White establishment to force the relocation of the Black community, within days of the *Label*, Black Tulsans had already begun rebuilding Greenwood. Thousands, however, were forced to spend the winter of 1921–22 living in tents. Moreover, for many years the *Label* became something of a taboo subject, particularly in Tulsa. One of the great tragedies of Oklahoma history, this eruption of bloodshed and destruction in Tulsa has lived on as a potent symbol of the ongoing struggle of Black and White Oklahomans to forge a common destiny out of an often troubled past.

Supplementary Materials S1: Summary article stimuli participants read in each valence framing condition.

Supplementary Table 1

Variable name	Description	Response option
violence	Rate the degree of violence attributable to each group's behavior during the Tulsa Race Massacre.	[1:7 – 1 Not at All, 4 Somewhat/Some, 7 Very Much]
blame	Rate the degree of blame attributable to each group's behavior during the Tulsa Race Massacre.	[1:7 – 1 Not at All, 4 Somewhat/Some, 7 Very Much]
caused	Rate the degree to which each group caused the Tulsa Race Massacre.	[1:7 – 1 Not at All, 4 Somewhat/Some, 7 Very Much]
intention	Rate the degree to which each group intentionally or deliberately brought about the Tulsa Race Massacre.	[1:7 – 1 Not at All, 4 Somewhat/Some, 7 Very Much]
punish	Rate the degree of punishment each group deserves for the Tulsa Race Massacre.	[1:7 – 1 Not at All, 4 Somewhat/Some, 7 Very Much]
allow-commit	Rate the degree to which each group allowed the Tulsa Race Massacre to happen or committed it.	[1:7 – 1 Allowed it to Happen, 4 Unsure or Neutral, 7 Committed the Action]
knew	Rate the degree to which each group knew the Tulsa Race Massacre would happen.	[1:7 – 1 Not at All, 4 Somewhat/Some, 7 Very Much]

should	Rate the degree to which each group should have prevented the Tulsa Race Massacre from happening.	[1:7 – 1 Not at All, 4 Somewhat/Some, 7 Very Much]
could	Rate the degree to which each group could have prevented the Tulsa Race Massacre from happening.	[1:7 – 1 Not at All, 4 Somewhat/Some, 7 Very Much]

Table 1: Rating items in order presented for both study cohorts.

Supplementary Table 2

	F value	Pr(>F)	
Framing	0.43	0.65	
Knowledge	2.92	0.09	.
Target	1614.33	<2e-16	***
Item	56.86	<2e-16	***
cohort	4.51	0.03	*
Framing:Knowledge	1.19	0.31	
Framing:Target	1.98	0.09	.
Framing:Item	0.85	0.63	
Knowledge:Target	117	<2e-16	***
Knowledge:Item	1.42	0.18	
Target:Item	54.27	<2e-16	***
Framing:Knowledge:Target	3.46	0.01	**
Framing:Knowledge:Item	0.65	0.84	
Framing:Target:Item	0.62	0.95	
Knowledge:Target:Item	3.98	<2e-16	***
Framing:Knowledge:Target:Item	0.79	0.79	

Signif. codes: 0 ‘***’ 0.001 ‘**’ 0.01 ‘*’ 0.05 ‘.’ 0.1 ‘ ’ 1

Table 2: ANOVA table of model results
